# Supplementary material for: Population genomics of ancient and modern Trichuris trichiura
Source: Nat Commun. 2022 Jul 6;13:3888. doi: 10.1038/s41467-022-31487-x (PMC9259628; doi:10.1038/s41467-022-31487-x)
Supplement: Supplementary file 1 — Supplementary Information [file 41467_2022_31487_MOESM1_ESM.pdf]

Supplementary Information

Population genomics of ancient and modern *Trichuris trichiura*

Stephen R. Doyle, Martin Jensen Søe, Peter Nejsum, Martha Betson, Philip J. Cooper, Lifei Peng, Xing-Quan Zhu, Ana Sanchez, Gabriela Matamoros, Gustavo Adolfo Fontecha Sandoval, Cristina Cutillas, Louis-Albert Tchuem Tchuenté, Zeleke Mekonnen, Shaali M. Ame, Harriet Namwanje, Bruno Levecke, Matthew Berriman, Brian Lund Fredensborg, Christian Moliin Outzen Kapel

List of Figures

- **Supplementary Fig. 1:** The estimated age of sampling sites from which ancient *Trichuris trichiura* samples were collected.
- **Supplementary Fig. 2:** Evidence of deamination in raw sequencing reads.
- **Supplementary Fig. 3:** Interaction between sequencing coverage and genetic diversity within samples per population.
- **Supplementary Fig. 4:** Parasite sex determination based on relative X-to-autosome sequencing coverage.
- **Supplementary Fig. 5:** Genetic analysis of leaf monkey and colobus monkey isolates.
- **Supplementary Fig. 6:** Extended admixture analysis.
- **Supplementary Fig. 7:** Kinship analysis.
- **Supplementary Fig. 8:** Extended treemix analysis.
- **Supplementary Fig. 9:** Comparison of private and shared variation between Ugandan, Chinese, and American isolates.
- **Supplementary Fig. 10:** Comparison of nucleotide diversity within each population.
- **Supplementary Fig. 11:** Comparison of nucleotide diversity between populations.
- **Supplementary Fig. 12:** Analysis of variation within and surrounding the β-tubulin gene.

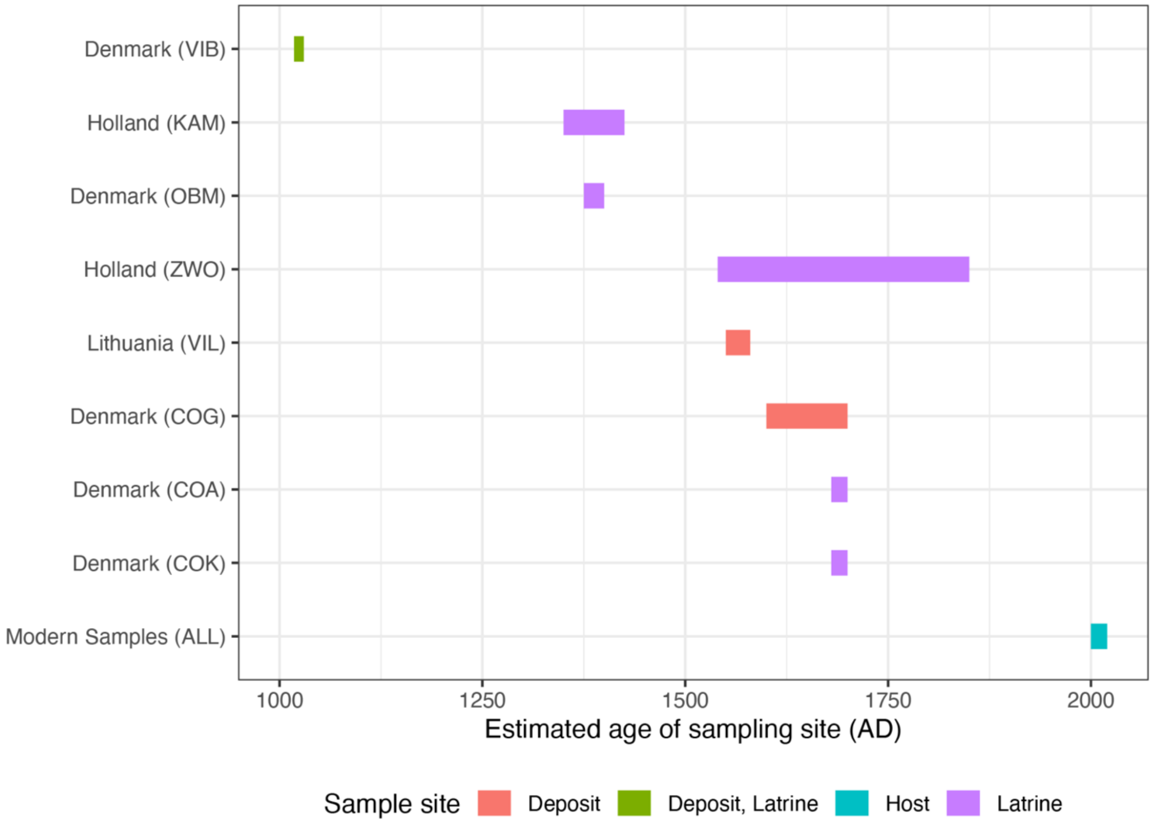

Supplementary Fig. 1: The estimated age of sampling sites from which ancient *Trichuris spp.* samples were collected.

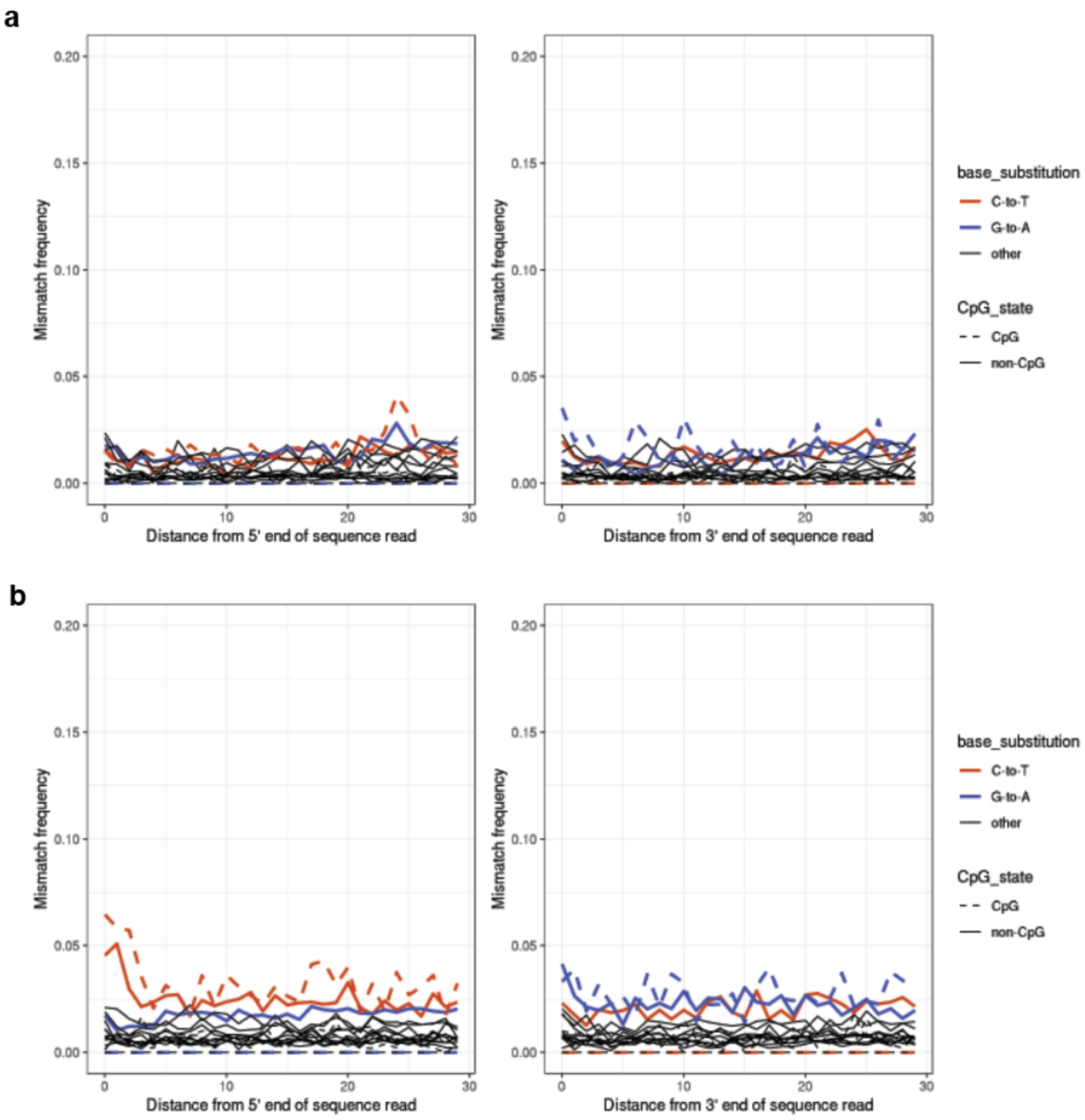

Supplementary Fig. 2: Evidence of deamination in raw sequencing reads. Deamination of cytosines is a common artefact in ancient DNA, evidenced by an increase in C-to-T and G-to-A substitutions. **a**. Example of a modern sample (MN_CHN_GUA_HS_001). **b**. Example of an ancient sample (AN_DNK_COG_EN_0012), showing an increase in C-to-T frequency at the beginning of the reads. To account for this, we removed the first two bases from all reads.

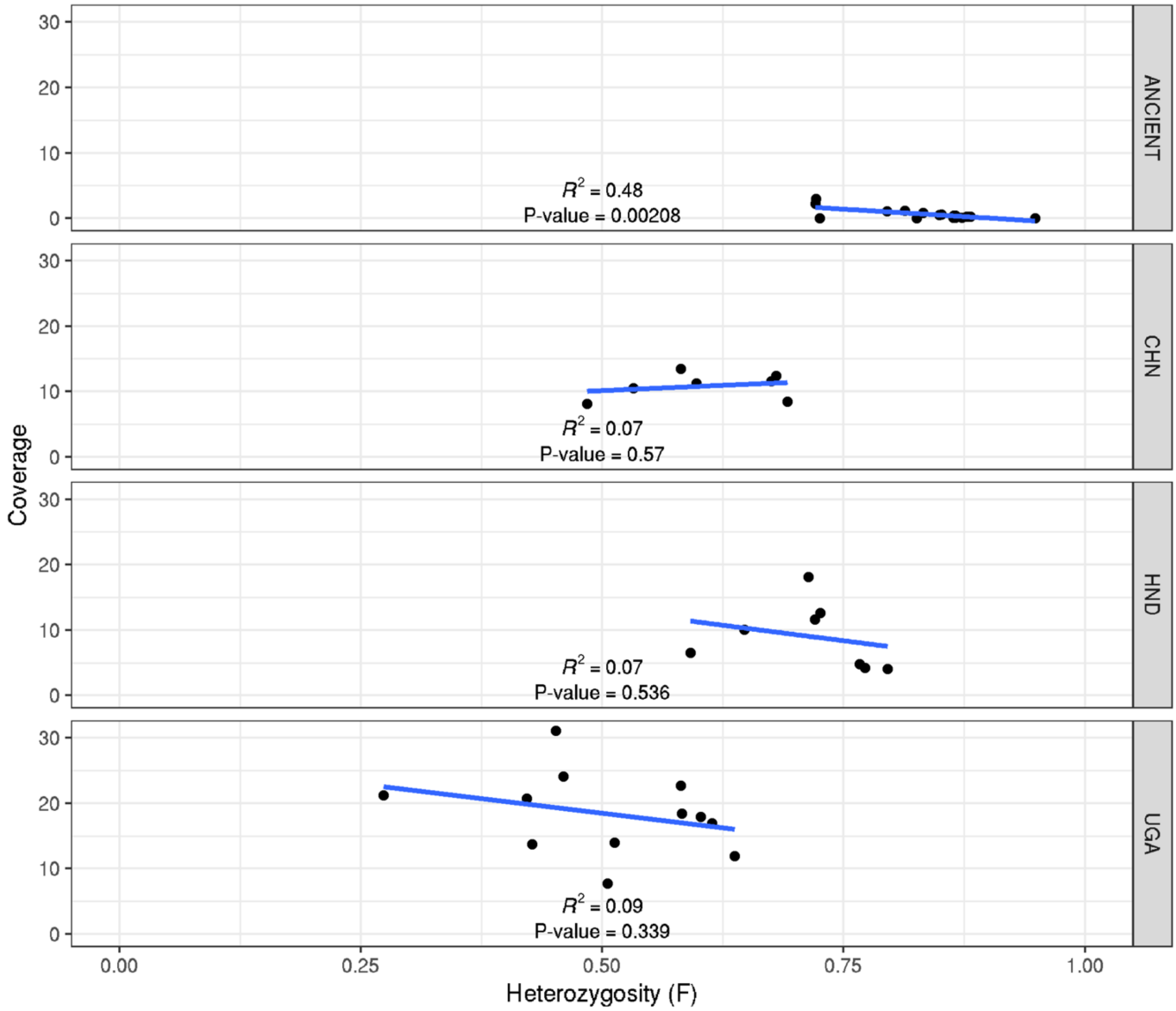

Supplementary Fig. 3: interaction between sequencing coverage and genetic diversity within samples per population. The analysis compares heterozygosity (x-axis) and sequencing coverage (y-axis) to determine if sequencing coverage biases estimates of genetic diversity. Each point represents an individual worm sample. The linear regression line, correlation coefficient (R^2) and p-value (cor.test, two-sided; adjusted p-value [“holm”]) testing the difference of the observed slope from 0 is shown.

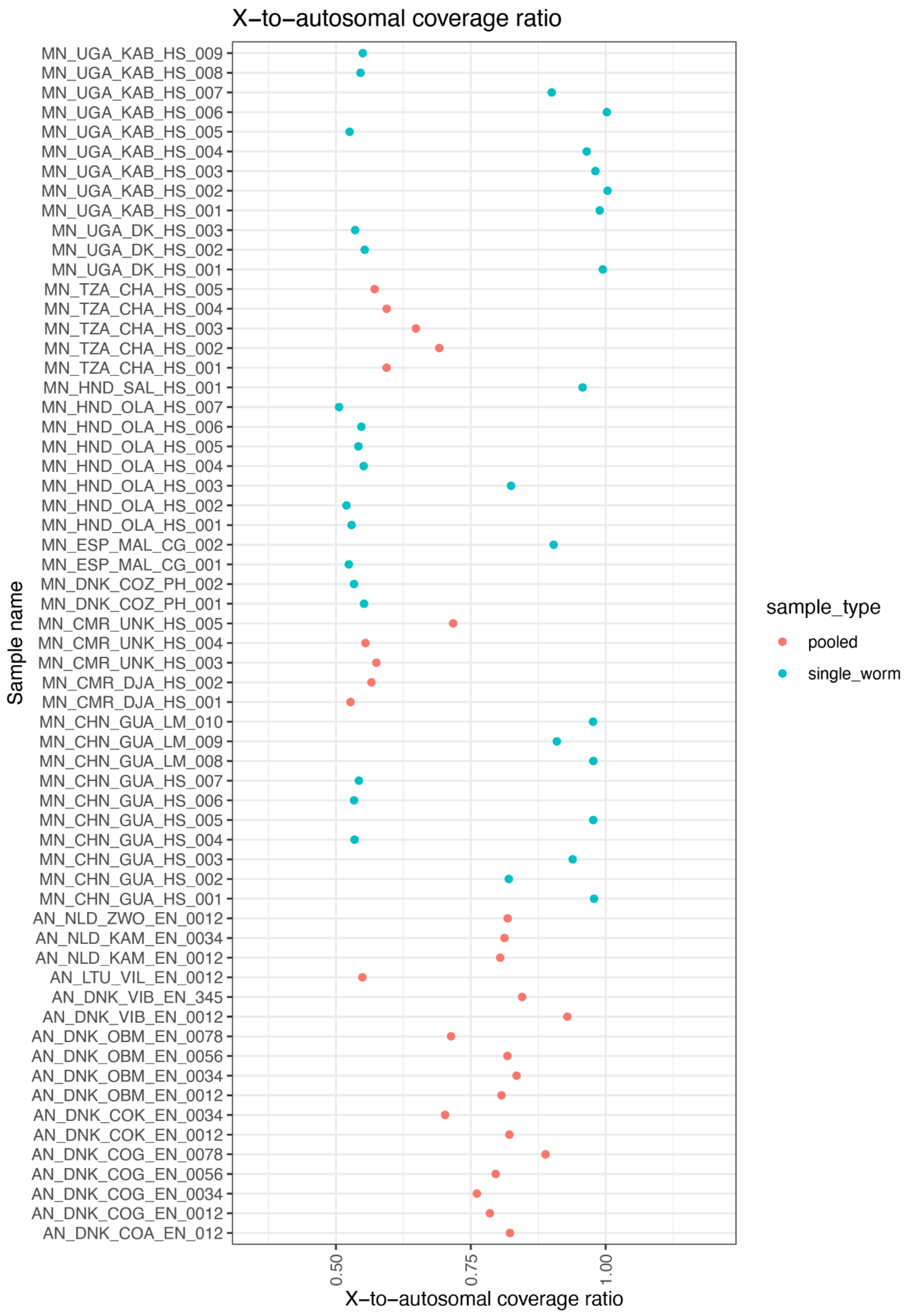

X–to–autosomal coverage ratio
MN_UGA_KAB_HS_009
MN_UGA_KAB_HS_008
MN_UGA_KAB_HS_007
MN_UGA_KAB_HS_006
MN_UGA_KAB_HS_005
MN_UGA_KAB_HS_004
MN_UGA_KAB_HS_003
MN_UGA_KAB_HS_002
MN_UGA_KAB_HS_001
MN_UGA_DK_HS_003
MN_UGA_DK_HS_002
MN_UGA_DK_HS_001
MN_TZA_CHA_HS_005
MN_TZA_CHA_HS_004
MN_TZA_CHA_HS_003
MN_TZA_CHA_HS_002
MN_TZA_CHA_HS_001
MN_HND_SAL_HS_001
MN_HND_OLA_HS_007
MN_HND_OLA_HS_006
MN_HND_OLA_HS_005
MN_HND_OLA_HS_004
MN_HND_OLA_HS_003
MN_HND_OLA_HS_002
MN_HND_OLA_HS_001
MN_ESP_MAL_CG_002
MN_ESP_MAL_CG_001
MN_DNK_COZ_PH_002
MN_DNK_COZ_PH_001
MN_CMR_UNK_HS_005
MN_CMR_UNK_HS_004
MN_CMR_UNK_HS_003
MN_CMR_DJA_HS_002
MN_CMR_DJA_HS_001
MN_CHN_GUA_LM_010
MN_CHN_GUA_LM_009
MN_CHN_GUA_LM_008
MN_CHN_GUA_HS_007
MN_CHN_GUA_HS_006
MN_CHN_GUA_HS_005
MN_CHN_GUA_HS_004
MN_CHN_GUA_HS_003
MN_CHN_GUA_HS_002
MN_CHN_GUA_HS_001
AN_NLD_ZWO_EN_0012
AN_NLD_KAM_EN_0034
AN_NLD_KAM_EN_0012
AN_LTU_VIL_EN_0012
AN_DNK_VIB_EN_345
AN_DNK_VIB_EN_0012
AN_DNK_OBM_EN_0078
AN_DNK_OBM_EN_0056
AN_DNK_OBM_EN_0034
AN_DNK_OBM_EN_0012
AN_DNK_COK_EN_0034
AN_DNK_COK_EN_0012
AN_DNK_COG_EN_0078
AN_DNK_COG_EN_0056
AN_DNK_COG_EN_0034
AN_DNK_COG_EN_0012
AN_DNK_COA_EN_012
Sample name
0.50
0.75
1.00
X–to–autosomal coverage ratio
sample_type
pooled
single_worm

Supplementary Fig. 4: Parasite sex determination based on relative X-to-autosome sequencing coverage. The mean coverage of the X-linked scaffolds (linkage group indicated by “Trichuris_trichiura_1” in reference) was compared with the mean coverage from the autosomal scaffolds (linkage groups “Trichuris_trichiura_2” and “Trichuris_trichiura_3” in reference). Male worm (XY) sex-linked scaffolds present at half-coverage of the autosomes (i.e., ratio = ~0.5), whereas female worms (XX) show equal coverage between sex-linked and autosomal scaffolds (i.e., ratio = ~1). Note that the ancient samples, as well as a subset of the African samples (Tanzania & Cameroon), are pools of eggs and so the ratio of male to females may vary per sample; if an equal sex ratio was present, we would expect a sex/autosome ratio of approximately 0.75.

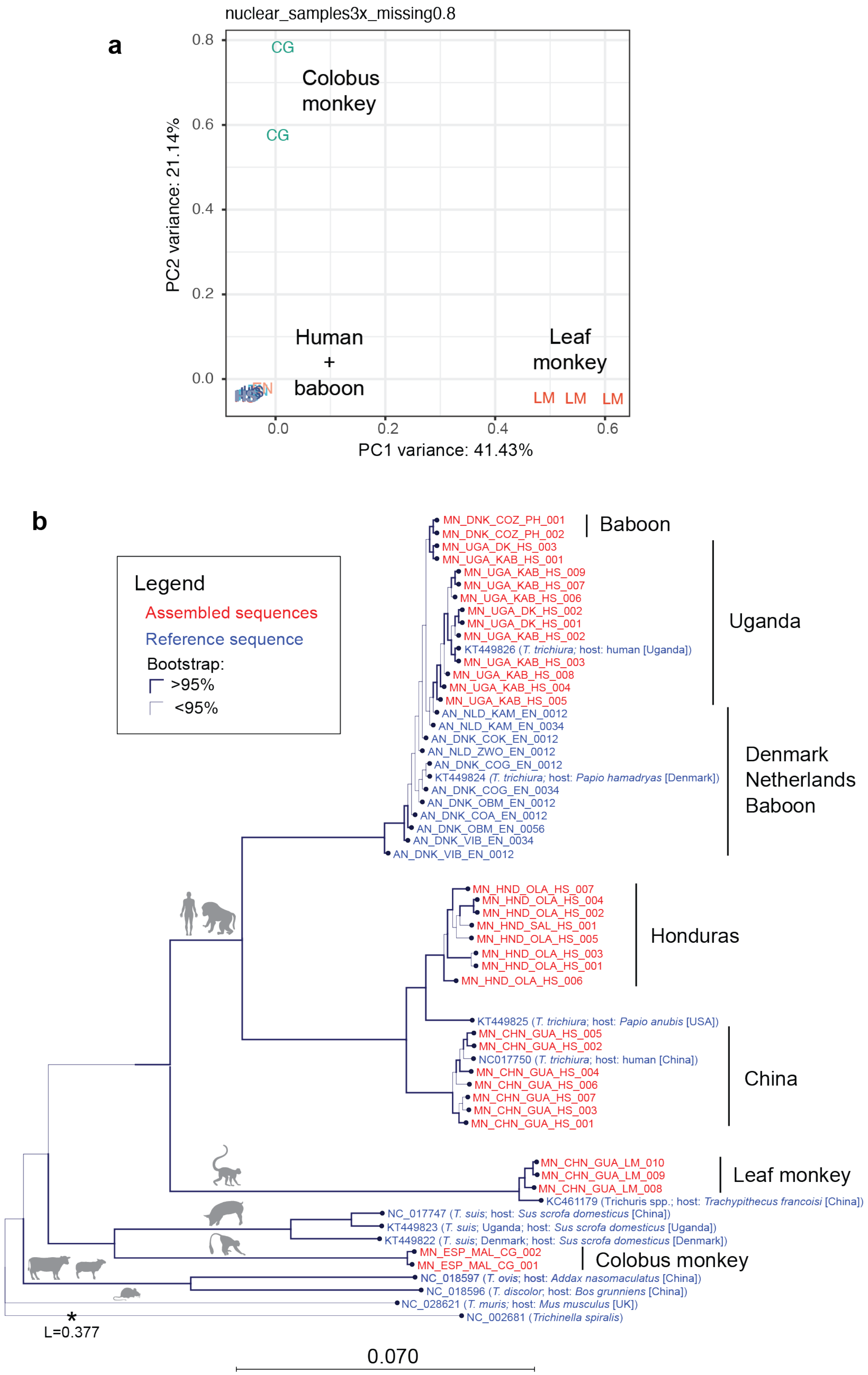
a
nuclear_samples3x_missing0.8
Colobus monkey
Human + baboon
Leaf monkey
CG
LM
PC2 variance: 21.14%
PC1 variance: 41.43%
b
Legend
Assembled sequences
Reference sequence
Bootstrap:
>95%
<95%
Baboon
Uganda
Denmark Netherlands Baboon
Honduras
China
Leaf monkey
Colobus monkey
KT449826 (T. trichiura; host: human [Uganda])
KT449824 (T. trichiura; host: Papio hamadryas [Denmark])
KT449825 (T. trichiura; host: Papio anubis [USA])
NC017750 (T. trichiura; host: human [China])
KC461179 (Trichuris spp.; host: Trachypithecus francoisi [China])
NC_017747 (T. suis; host: Sus scrofa domesticus [China])
KT449823 (T. suis; Uganda; host: Sus scrofa domesticus [Uganda])
KT449822 (T. suis; Denmark; host: Sus scrofa domesticus [Denmark])
NC_018597 (T. ovis; host: Addax nasomaculatus [China])
NC_018596 (T. discolor; host: Bos grunniens [China])
NC_028621 (T. muris; host: Mus musculus [UK])
NC_002681 (Trichinella spiralis)
L=0.377
0.070

Supplementary Fig. 5: Genetic analysis of leaf monkey and colobus monkey isolates. a. PCA of nuclear variants, including highlighting genetically distinct colobus and leaf monkey samples from the closely related, indistinguishable cluster of human and baboon samples. **b.** Neighbour-joining phylogeny of assembled mitochondrial genomes (red) together with publicly available whole mitochondrial genome data (blue; note some of the ancient mitochondrial genomes had previously been described by Søe et al. 2018) for species within the *Trichuris* genus. *Trichinella spiralis* was used as an outgroup. Branch lengths less than 0.002 are set to 0.002.

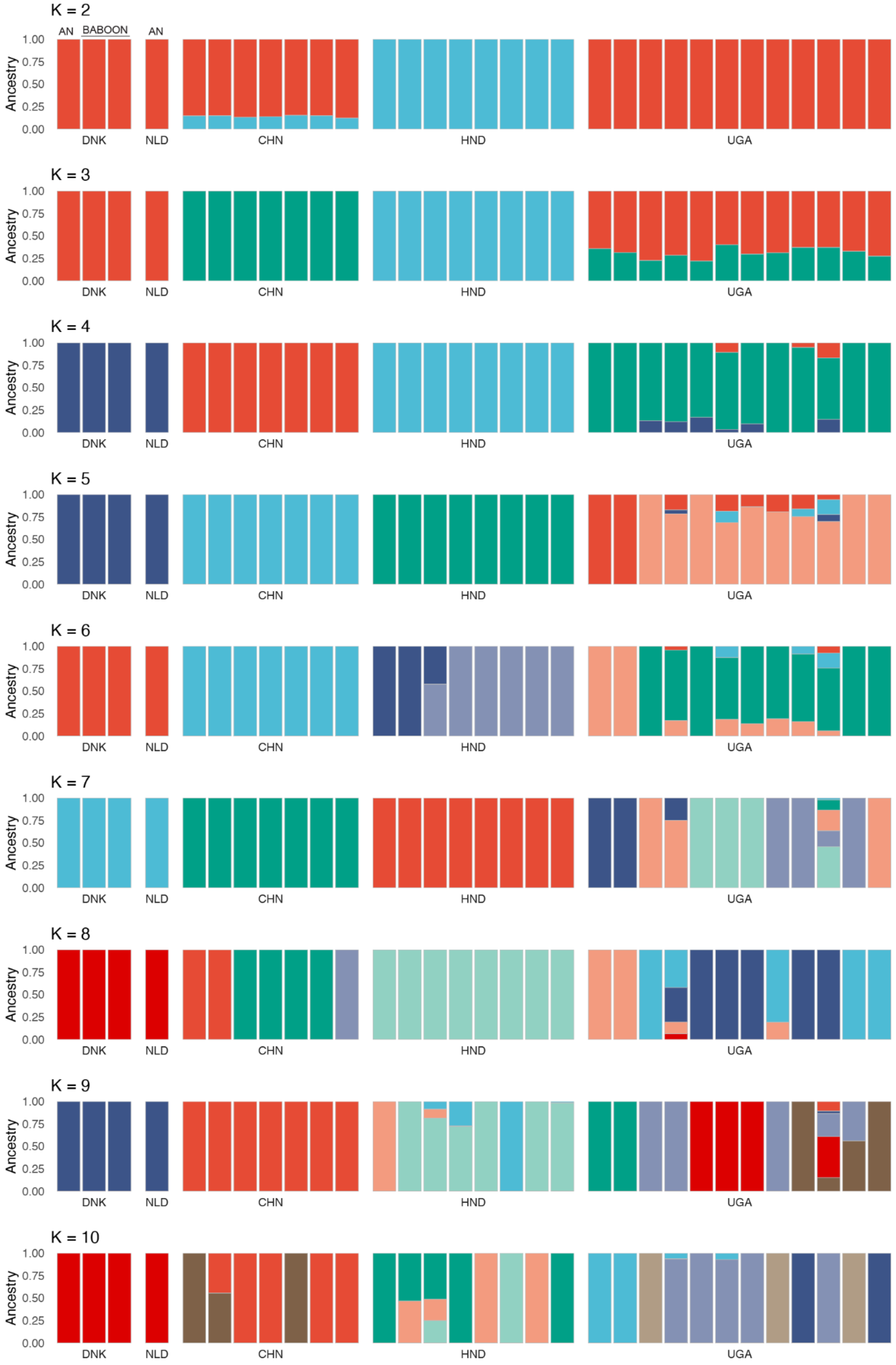

K = 2
AN
BABOON
AN
1.00
0.75
0.50
0.25
0.00
Ancestry
DNK
NLD
CHN
HND
UGA
K = 3
K = 4
K = 5
K = 6
K = 7
K = 8
K = 9
K = 10

Supplementary Fig. 6: Extended admixture analysis. Analysis of genetic admixture between samples (individual bars; n = 39) and populations (x-axis groups) across a range of theoretical ancestral genetic populations (individual colours) ranging from k = 2 to k = 10 using 857,169 nuclear variants. Ancient samples are represented as the first DNK sample and the NLD sample, and the baboon samples are represented as the second and third DNK samples. The remaining samples are modern from each respective country.

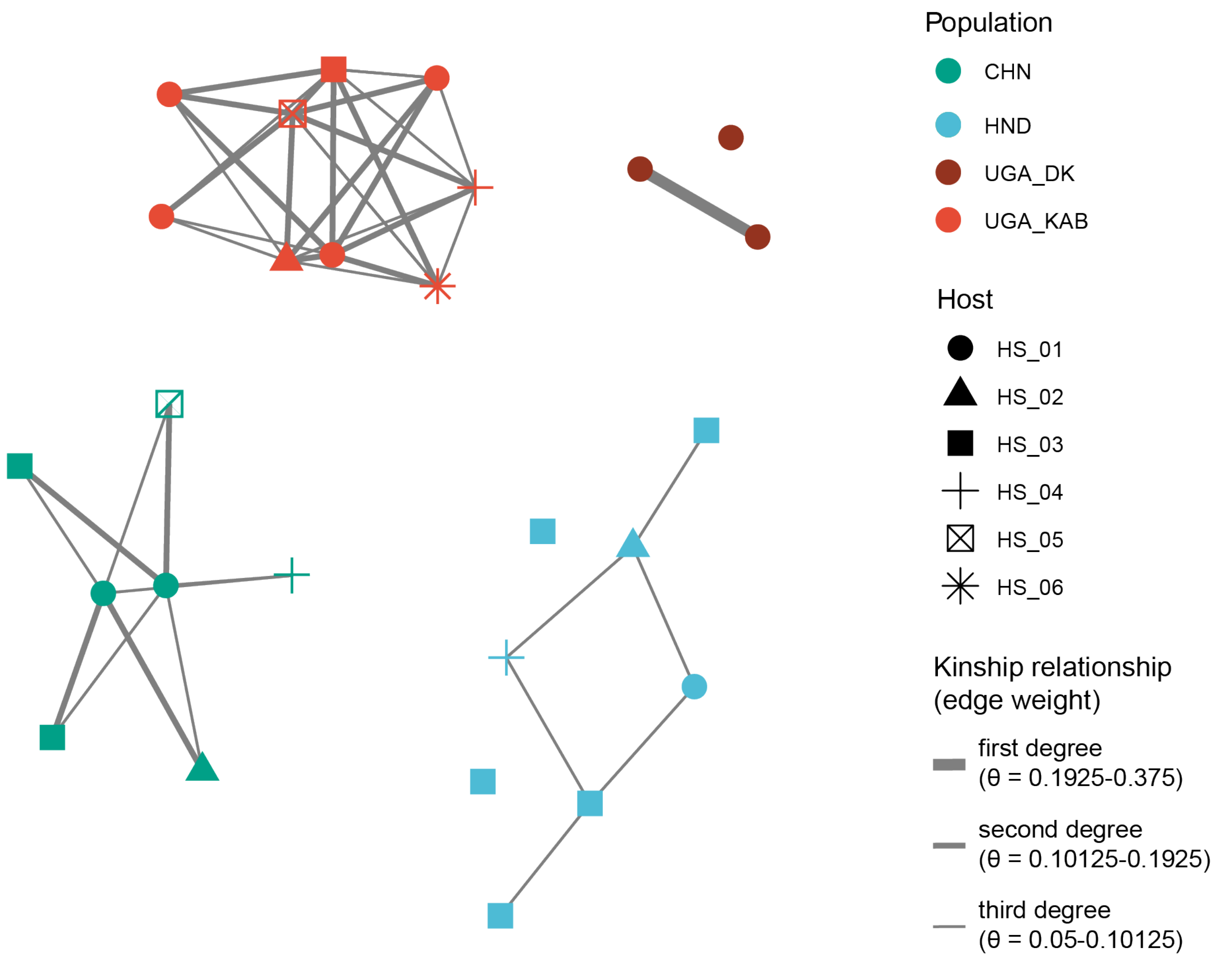

Supplementary Fig. 7: Kinship analysis. Pairwise estimates of kinship were determined within each population for samples with sufficient sequencing coverage. Each point represents an individual worm and is coloured by the population from which it was sampled. Uganda samples (UGA_DK & UGA_KAB) are intentionally split due to the difference in sampling, as UGA_DK samples were obtained from a controlled infection of a single host. Sample shapes indicate if worms were sampled from the same or different host. The edge lines linking samples indicate the degree of relatedness between the samples. Although many inferred relationships are shown by connecting edges, most are between distinct hosts which without knowing the host relationships (all deidentified in this study), it is difficult to know if these are true kinship relationships between worms or not. On the other hand, the single first-degree relationship between UGA_DK samples is likely true, given these worms were sampled from the same host.

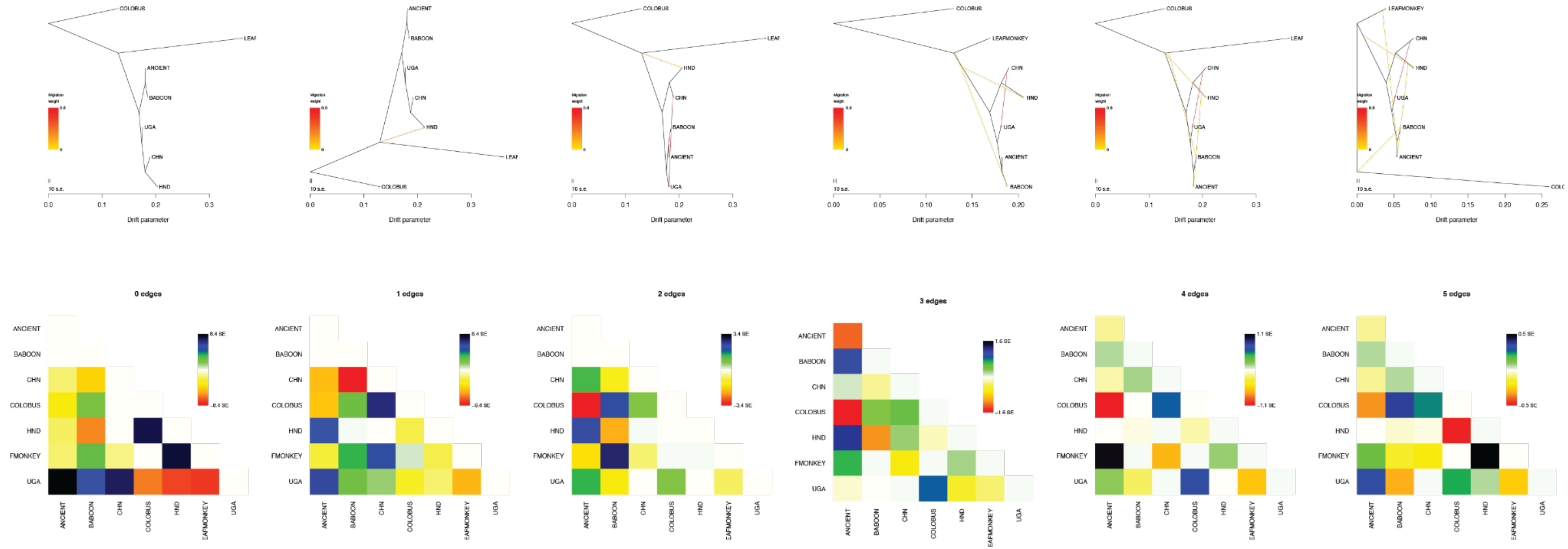

Supplementary Fig. 8: Extended treemix analysis. Treemix maximum likelihood trees of ancient and modern samples, including Colobus and Leaf monkey samples as outgroups, across a range of hypothetical migration edges that range from no migration events (m = 0 edges) to five migration events (m = 5 edges). The support for those migration edges - the migration weight - is represented by the coloured line joining populations. We also present matrices of the pairwise residuals below each tree. Positive residual values between pairs of populations indicate that they are more closely related to each other than is inferred by the best-fit tree, and therefore, provide a finer-grained view of potential admixture events.

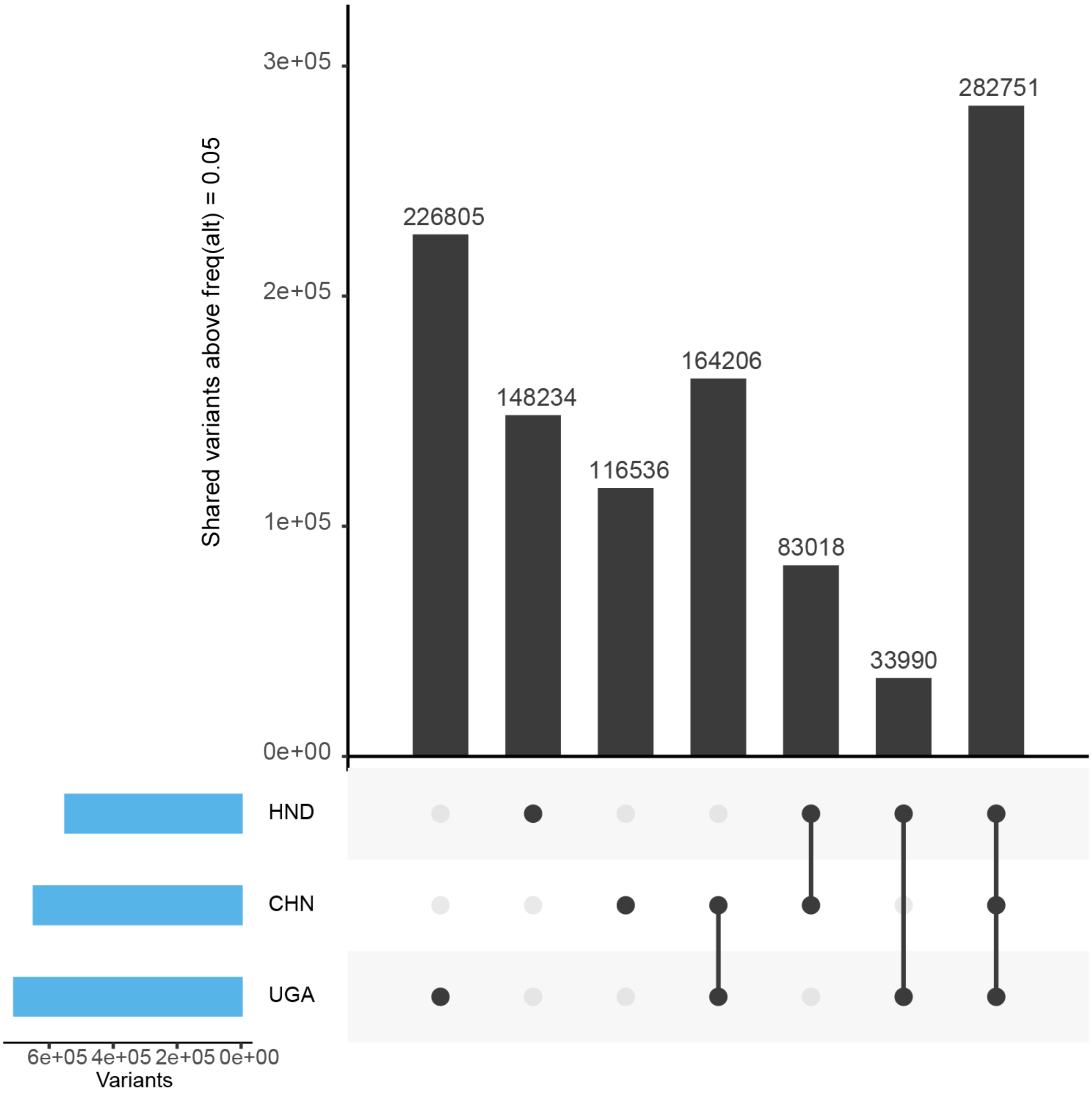

Supplementary Fig. 9: Comparison of private and shared variation between Ugandan, Chinese, and Honduran isolates. Nuclear variants were subset into groups depending on whether they were presented in a single population (ie. private), shared by only two populations, or by all three populations. The total number of variants per group is shown (black vertical bars). The total number of variants per country, summed across all comparisons, is also shown (blue horizontal bars). Given the step-wise hypothesis by which humans migrated from Africa to Asia and then subsequently to the Americas, the rationale for this analysis was to identify the proportion of variation shared by Ugandan and Honduran populations that is not present in the Chinese population, which may indicate independent translocation.

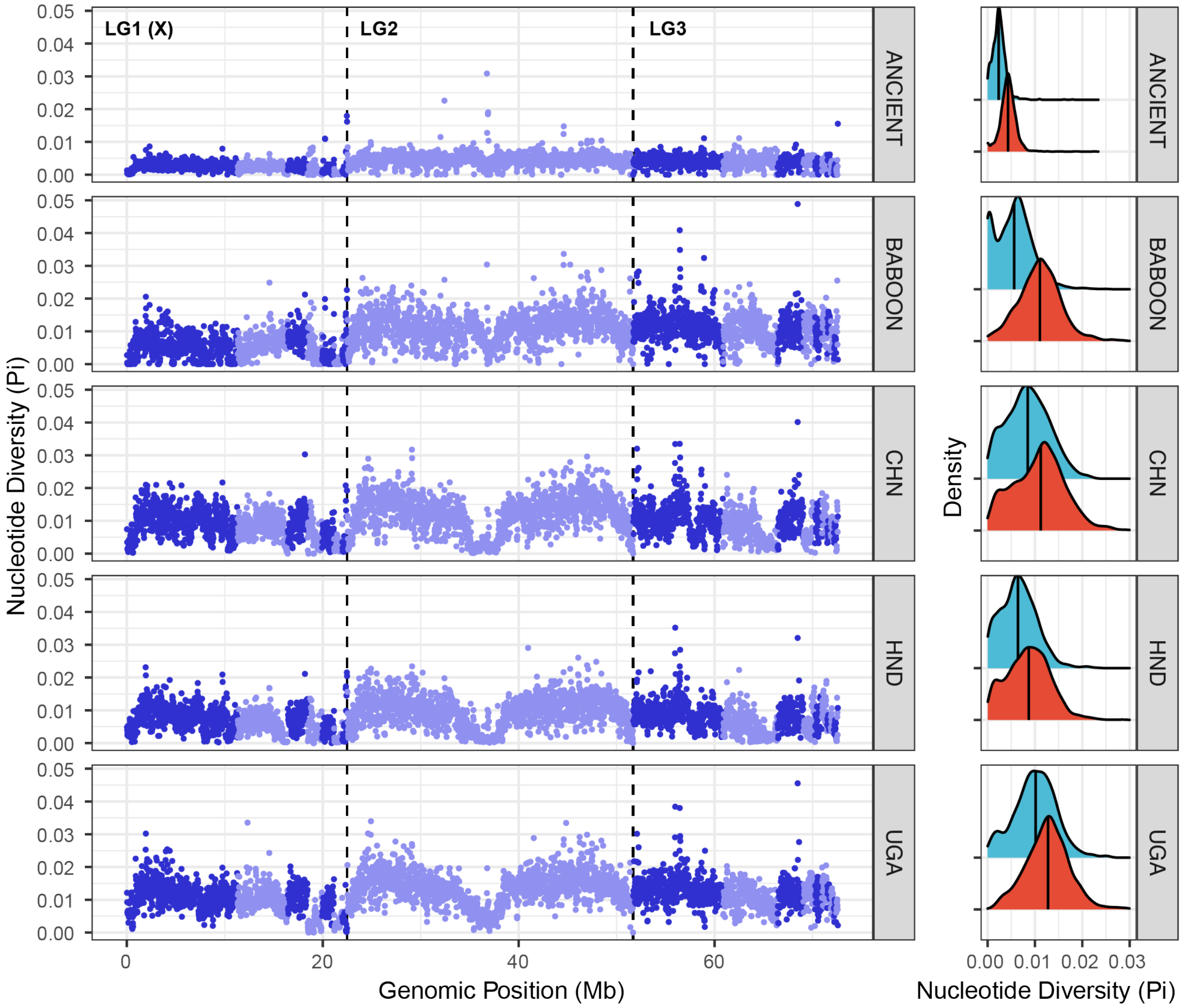

Supplementary Fig. 10: Comparison of nucleotide diversity within each population. Comparison of genetic differences throughout the genome between human-infective *T. trichiura* from genetically and geographically defined populations. Nucleotide diversity was measured in 20 kb windows for each population. Alternating dark and light blue colours represent different scaffolds, with the vertical dashed lines separating the three chromosomal linkage groups (LG). The sex-linked linkage group is indicated as LG1 (X). Sample abbreviations: CHN=China; UGA=Uganda; HND=Honduras. Density plots show the distribution of π values for the sex-linked (blue) and autosomal (red) scaffolds. The median π value for each is shown (solid vertical line).

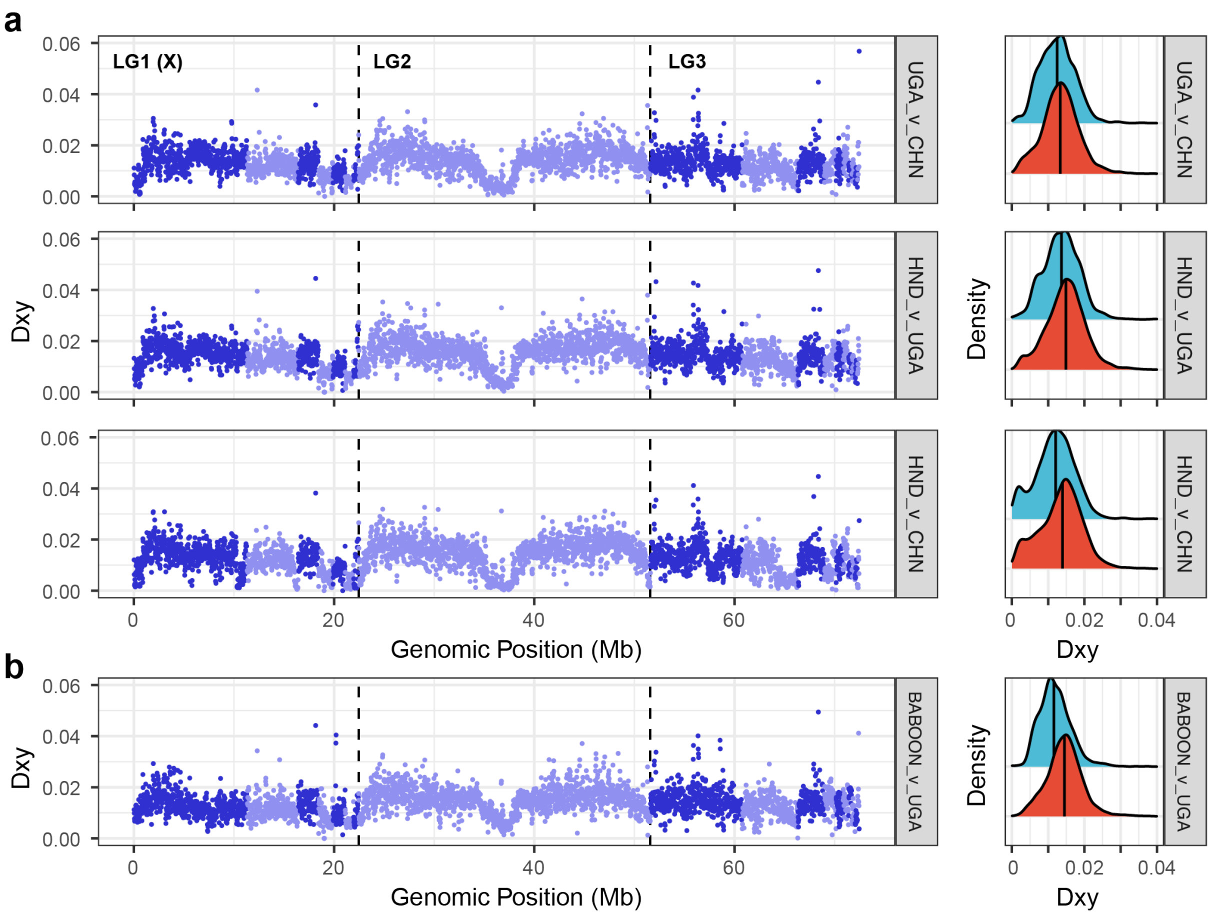

Supplementary Fig. 11: Comparison of nucleotide diversity between populations. a. Comparison of genetic differences throughout the genome between human-infective *T. trichiura* from genetically and geographically defined populations. Pairwise *D*xy was measured in 20 kb windows between China and Uganda (top), Uganda and Honduras (middle), and China and Honduras (bottom). Alternating dark and light blue colours represent different scaffolds, with the vertical dashed lines separating the three chromosomal linkage groups (LG). The sex-linked linkage group is indicated as LG1 (X). **b.** Comparison of genome-wide differences between the closely related human-infective Ugandan and baboon-infective *T. trichiura*. Sample abbreviations: CHN=China; UGA=Uganda; HND=Honduras. Density plots in a and b show the distribution of *D*xy values for the sex-linked (blue) and autosomal (red) scaffolds. The median *D*xy value for each is shown (solid vertical line).

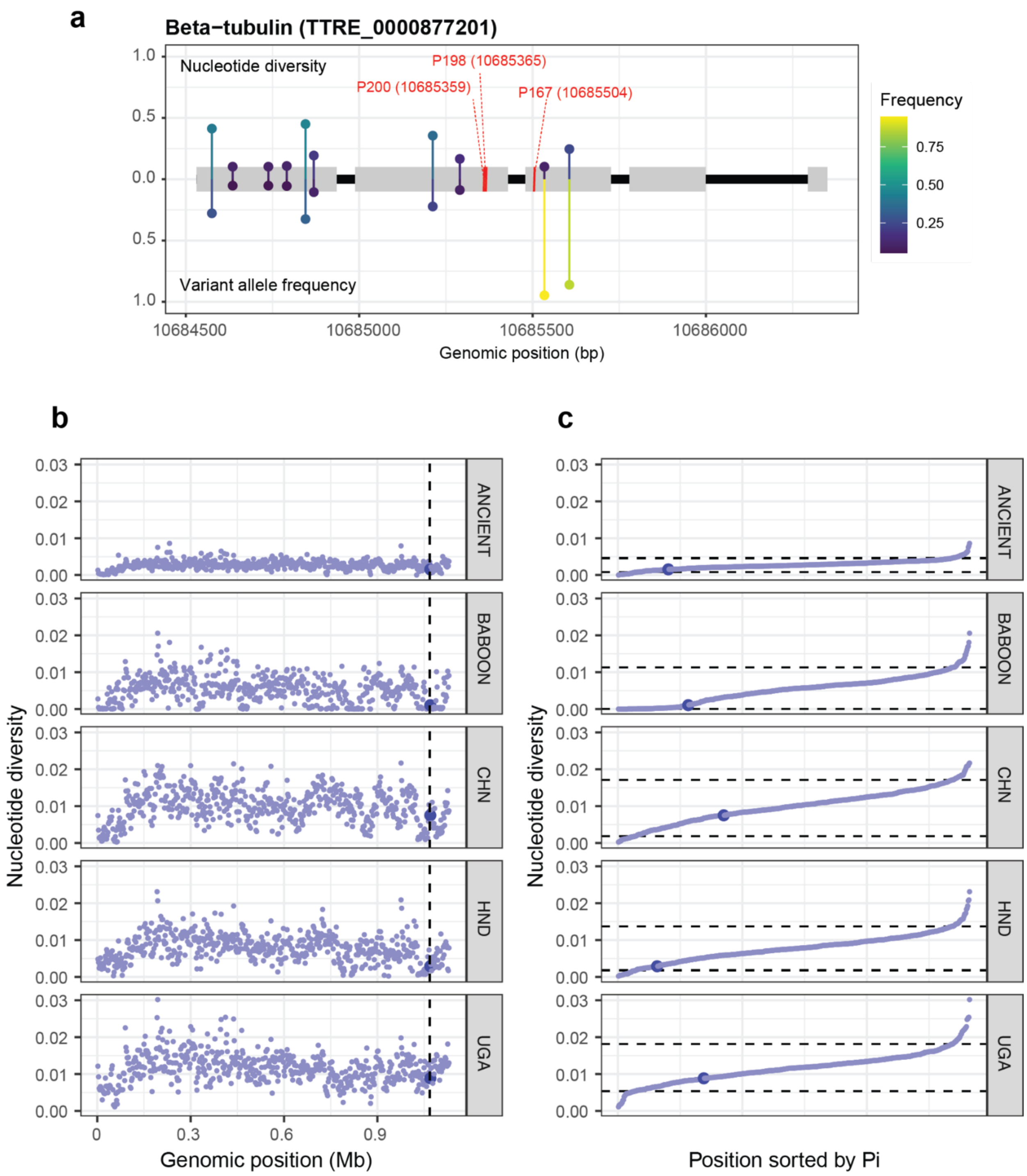

Supplementary Fig. 12: Analysis of variation within and surrounding the β-tubulin gene. a. Gene structure of the β-tubulin gene (TTRE_0000877201), with exons presented as grey boxes separated by black lines representing introns. Variants identified in the global cohort are indicated by coloured lines; above the gene mode, the nucleotide diversity at each position is shown, whereas, below the gene model, the variant allele frequency is shown. Both are coloured by their relative frequencies. The red lines represent the positions of canonical resistance-associated variants—P167, P198, and P200 (actual position of the

codon in gene model is indicated in parentheses)—at which no variation was identified here. **b.** Nucleotide diversity (measured in 20 kb non-overlapping windows; light blue point) in the Trichuris_trichiura_1_001 scaffold containing the β-tubulin gene, indicated by the black vertical line and dark blue point, in each population. **c**. Ranked order of nucleotide diversity values shown in b. The upper and lower dashed lines represent the 95th and 5th quantiles of the distribution, respectively. The β-tubulin gene (dark blue point) is highlighted, demonstrating its level of nucleotide diversity is not deemed to be an outlier relative to the observed diversity throughout the scaffold.
