# Supplementary material for: Population genomics of ancient and modern Trichuris trichiura
Source: Nat Commun. 2022 Jul 6;13:3888. doi: 10.1038/s41467-022-31487-x (PMC9259628; doi:10.1038/s41467-022-31487-x)
Supplement: Supplementary file 3 — Description of Additional Supplementary Files [file 41467_2022_31487_MOESM3_ESM.pdf]

Description of Additional Supplementary Files

File Name: Supplementary Data 1
Description: Sample metadata and sequencing accession numbers.

File Name: Supplementary Data 2
Description: Genome mapping data by sample, including nuclear and mitochondrial genome coverage, and deamination statistics.

File Name: Supplementary Data 3
Description: Description of genes in regions of high genetic differentiation between samples from China and Uganda.

File Name: Supplementary Data 4
Description: Description of genes in regions of high genetic differentiation between samples from Uganda and Honduras.

File Name: Supplementary Data 5
Description: Description of genes in regions of high genetic differentiation between samples from China and Honduras.

File Name: Supplementary Data 6
Description: Description of genes in regions of high genetic differentiation between samples from Baboons and Uganda.
